# Anæmia, Leukæmia, and Lymphadenoma

**Published:** 1895-12-21

**Authors:** 


### Progress in Medicine.

### ANÆMIA, LEUKÆMIA, AND LYMPHADENOMA.

**Anæmia.**—As our knowledge of these diseases increases the hope of a simpler classification grows stronger. Chlorosis, splenic anæmia, pernicious anæmia, lymphadenoma, some seven varieties of leucocythæmia, and many other divisions perplex the student, while the treatment of most forms is unsatisfactory, and is but an echo of our ignorance of their true pathology. However, some advance has been made. Thus, R. Stockman[1] of late has shown strong grounds for believing that pernicious anæmia is only a high degree of common anæmia starting from various causes, and that only when persistent and oft-repeated capillary hæmorrhages have occurred the progressive fatal type is formed. Thus dyspepsia, malignant growths, parasitic worms, and fevers, or great external hæmorrhage may so damage the vessels as to lead to these recurring capillary hæmorrhages, which in turn cause a deposit of iron in many organs as well as the liver. These deposits, too, can be produced experimentally, and are found also in purpura, but are absent where the persistent hæmorrhage is an external one, such as that which the ankylostoma causes. This appears a fair argument against the theory that the essence of the disease consists in some destructive agent acting on the blood corpuscles in the liver. The view that the disease is due to deficient secretion by the bone marrow, just as myxœdema depends on thyroid atrophy, has little foundation. The parallel is rather with Graves' disease, since we have at least hypertrophy of the marrow; and probably, after all, if Stockman is right, this hypertrophy is only evidence of the attempted repair against the constant loss of blood corpuscles. The question of treatment is a difficult one. Arsenic often initiates improvement, though it may fail to maintain it; Stockman and others insist on the need of iron. Bone marrow, given raw in bread-crumbs, wine, or milk, seems to give most uncertain results. A few remarkable recoveries are recorded. Recently Barrs related a successful case where arsenic caused symptoms of poisoning, but the administration of three ounces daily of red bone marrow for a long period was followed by complete cure. One other case of apparently pernicious anæmia, under the care of W. B. Drummond, recovered under the same treatment.[2] The success of Frazer and one or two others in the use of marrow has not been attained generally, and its mode of action is not yet understood.

Chlorosis is a type of anæmia which presents the advantage of yielding easily to treatment. Whatever is our theory of its cause and the action of remedies upon it, rest in bed with iron rapidly cures most of the uncomplicated cases. It has been asserted that the disease is due to intestinal fermentation; that it is merely part of defective development and associated with a small heart or an ill-formed pelvis[3] and an infantile condition generally; and recently the suggestion has been made that the adolescent female stores up iron in her tissues in preparation for pregnancy, to her own impoverishment. Even the infant lives for a long time on the iron supplied in intra-uterine life, for milk contains a very inadequate supply. At the German Congress of Physicians the old controversy as to the absorption of iron when given as a drug was again threshed out. Bunge pointed out that over and over again when iron had been administered, the whole amount so given could be collected from the fæces, and no increase or change in that excreted in the urine occurs during the treatment. Practically he denied the value of ferruginous drugs, since the small amount which is needed by the organism is assimilated solely from organic sources, and hence recourse must be had to the products of the market garden rather than to the chemist. Vegetables and certain animal substances, such as the yolk of eggs, where iron is combined with a nucleo-albumen, are the products which afford iron to the body. But it has been shown, on the other hand, that the improvement following the use of Blaud's pills, for example, is so constant, ceasing if they are given up prematurely, and returning if they are again administered, that there can be no doubt whatever of their efficacy. In short, there must be some explanation of this curious discrepancy between the experimental and clinical evidence. Possibly, as Quincke suggested in the debate, the experiments were made on healthy individuals whose bodies did not need, and therefore did not absorb, iron; while anæmic organisms can and do take what they require. Another suggestion is that iron-containing drugs combine with the sulphuretted hydrogen of the intestine and prevent its poisonous action on the blood, a reaction which we may describe as antitoxic, and akin to the practice of washing out the intestines by saline purgatives so strongly recommended by Sir Andrew Clark. Some of the disbelievers in absorption go so far as to say that iron is injurious in chlorosis by producing dyspepsia. This, however, is common enough in the patients before any iron whatever has been administered, and though in some cases it is better to commence treatment by regulating digestion, yet in moderate gastric catarrh Von Ziemssen and other authorities hold that better results are obtained by giving iron at once rather than by waiting for the cure of the dyspepsia. Kobert,[4] however, appears to have answered the question as to the possibility of assimilating iron. He prepared a compound of pyrogallol with the iron contained in ox blood, and found that this substance, which he called hæmogallol, was easily absorbed without irritation, and also that the firmly combined iron in the urine was largely increased during its administration, showing that it was really assimilated. Friedburg and many others have reported remarkable results in the use of this compound, and a list of German writers on the subject is given in the American M. S. Bulletin of March 1st. Iron, too, has been demonstrated in the villi of the intestines after it was given with the food, and Marfori[5] found that another iron compound, ferratin, could be absorbed to the amount of 40 per cent., but only when the putrefactive processes of the intestines had been reduced by saline purgation and like measures. Potain and Hayem also protest against Bunge's theory,[6] and the latter recommends prolonged rest in bed and the protoxalate of iron, though if gastric dilatation and hyperpepsia exist he first puts the patient on a meat and milk diet. In some cases it will be necessary to wash out the stomach, but if merely hyperpepsia exists the iron may be given at once, and hydrochloric acid after food. Huchard,[7] too, thinks it important to treat the special form of dyspepsia before giving iron, and prefers to give proto salts; while Von Noorden agrees as to the value of iron, but holds that when absorbed it is really stored away in the liver and other tissues, and that it there exerts a stimulating or irritant effect on the blood-forming organs. Finally, in a recent analysis of 63 cases of chlorosis by Stockman,[8] it was noted that amendment occurred first in the number of the corpuscles, while the increase of hæmoglobin took place later on. This result gives some little support to Von Noorden's view. Moreover, he, too, found that the improvement only took place when iron was given, and he regards as immaterial the particular kind employed. It seems clear, that whatever our views of the pathology are, ferruginous treatment—used with care—produces satisfactory results.

**Lymphadenoma and Leukæmia.**—A decided advance has been made with regard to the former affection by its reproduction in a dog by inoculations of a pure culture of bacillus. This bacillus was found in the spleen of a patient affected with the disease, and after large injections of the culture had been given new growths appeared in the glands of the animal. From the new growths later on fresh cultures of the same bacillus were obtained by P. Delbet,[9] to whom the honour of the discovery belongs. Leucocythæmia has been also reproduced in a similar manner. The importance of the fact depends not only on the hitherto unknown origin of the disease, but on its close relation to sarcoma and other malignant growths. Thus an interesting discussion took place at the British Association meeting, chiefly on the question whether lymphadenoma is to be considered a granuloma, the result of some infective irritation, or a true malignant growth. It was pointed out that the growths, unlike sarcoma, do not infiltrate neighbouring organs, and can be shelled out of their enveloping membranes, and that occasionally a virulent form fatal in a few days is met with. Just as general tuberculosis and local tuberculous tumours occur, so, according to W. G. Spencer, we may have lymphadenomatosis or merely lymphomas. Leucocythæmia, he thinks, is only one symptom of the former state. Thus we seem in Delbet's bacillus to have a causal link between several diseases as different as phthisis, strumous glands and joints, for which Koch's bacillus is the common cause. There is reason to think that sometimes the poison gains entrance through the stomach, and at others through the lungs, and one remarkable case exists of a surgeon who was infected and killed after plugging the nostrils of a patient suffering from lymphadenoma. Delbet, indeed, seems to show that the system is able to destroy small doses of the poison in health, which is, of course, the case also with Koch's bacillus. The cases of acute leucocythæmia described by Fränkel, Benda, Heubner,[10] and others, often running their course in a few days, may present little or no splenic or glandular enlargement, though in other respects they seem to be true cases of the disease. Fränkel noticed that when a septic fever attacks a leucocythæmic patient there is an immense destruction of white corpuscles, and the same result has been attained by the injection of splenic extract or nuclein, but without any improvement in the general state. If Delbet's discovery is confirmed, probably some satisfactory method of treatment for those diseases may be found and the true relation between lymphadenoma and the various forms of leucocythemia will become clear. Meanwhile, the phenomena of pneumonia and other infective disorders are throwing much light on the multiplication and movements of the eosinophilous corpuscles, which strengthen the view that in leucocythæmia the organism has to struggle against some infective poison. Here fever of a peculiar type may be produced; glandular enlargements, splenic engorgement, and variation of the specially multiplied corpuscles may occur, according to the stage or acuteness of the disease,—a state of things which is closely parallel to what is seen in malaria and some infective fevers, though the new cells in the latter are indeed milti-nuclear, and in leucocythæmia mononuclear, or eosin granulavones. On the other hand, there are many analogies to the progress of malignant growths in which, to say the least, any infective origin is quite unproved.

[1] B.M.J., May 4, 11, 18. [2] B.M.J. Ep., May 18. [3] Practitioner, May. [4] Med. Rec., Oct. 26. [5] B.M.J., July 27. [6] An. T. Med. Sc., Sept. [7] Int. Med. Mag., July. [8] Ed. Med. J., Nov. [9] Med. Week, June 28. [10] Med. Week, June 8, 21.

